# Handwashing as a socially co-produced practice: an analysis through cultural-historical activity theory

**DOI:** 10.3389/fpubh.2026.1900210

**Published:** 2026-08-11

**Authors:** Munguntuul Enkhbat, Khandmaa Sukhbaatar, Buyandelger Batmunkh

**Affiliations:** 1 Department of Public Health Nursing, School of Nursing, Mongolian National University of Medical Sciences, Ulaanbaatar, Mongolia; 2 Division for Faculty and Students Development, Mongolian National University of Medical Sciences, Ulaanbaatar, Mongolia; 3 Department of Basic Science, School of Nursing, Mongolian National University of Medical Sciences, Ulaanbaatar, Mongolia

**Keywords:** activity system, culture, handwashing, health behavior, history of health

## Abstract

**Introduction:**

Handwashing is widely promoted as a key preventive behavior in public health; however, its historical development reflects a complex interplay of cultural, religious, and scientific influences that remain insufficiently theorized in the literature. This study examines the evolution of handwashing practices through the lens of Cultural-Historical Activity Theory, aiming to conceptualize handwashing as a product of interacting activity systems rather than a purely biomedical behavior.

**Methods:**

Drawing on a historical research design, we synthesized evidence from diverse cultural traditions, major religious texts, and the development of medical science from ancient civilizations to the modern era.

**Results:**

The analysis identified three interrelated activity systems (cultural, spiritual-religious, and medical-scientific) each characterized by distinct objects, norms, tools, and meanings that shaped handwashing practices over time. We show that persistent tensions and misalignments between these systems have influenced both the diffusion and the inconsistent adoption of handwashing practices in contemporary settings.

**Conclusion:**

These findings suggest that current public health approaches, which predominantly emphasize knowledge and infrastructure, may overlook the historically embedded, symbolic, and normative dimensions of hygiene behavior. We argue that integrating cultural and religious logic into hand hygiene interventions can enhance their effectiveness and sustainability. Reconceptualizing handwashing as a socially and historically co-produced practice offers a more comprehensive framework for designing context-sensitive public health strategies.

## 1 Introduction

Handwashing is widely recognized as one of the most effective measures for preventing infectious diseases and promoting public health (1, 2). Despite extensive global efforts to improve handwashing practices, particularly following the COVID-19 pandemic, significant gaps persist in both access to facilities and consistent adherence to recommended practices across different social and institutional contexts (3–5). These challenges are often framed in terms of insufficient knowledge, limited infrastructure, or behavioral noncompliance, which have been identified as major determinants influencing handwashing practices worldwide (6–8). However, such explanations may overlook the deeper historical and social processes through which handwashing practices were formed and sustained.

Rather than being solely a biomedical or technical behavior, handwashing can be understood as a socially co-produced practice shaped by the long-term interaction of cultural norms, religious beliefs, and scientific knowledge systems. Throughout historical periods, handwashing has served multiple and sometimes overlapping purposes, including maintaining bodily cleanliness, expressing moral and spiritual purity, and preventing disease transmission (9). These diverse meanings suggest that contemporary handwashing practices cannot be fully explained without considering the historical layering and interaction of these distinct, yet interconnected, domains.

Historical studies of hygiene have demonstrated that practices related to washing hands and bodily cleansing have existed across diverse civilizations, although their meanings and functions have varied according to social, cultural, and intellectual contexts (10–12). In many ancient societies, handwashing was associated with ritual preparation, social etiquette, and concepts of purity, whereas later developments in medical science gradually transformed hand hygiene into a practice primarily linked with infection prevention (10, 13, 14). The emergence of germ theory and modern microbiology further reshaped handwashing by establishing its role as a scientifically justified intervention for reducing disease transmission (15, 16). These historical transitions indicate that handwashing has not developed through a single linear pathway but through the continuous interaction of multiple systems of knowledge and practice.

Previous research has documented the importance of cultural context in shaping health behaviors and highlighted the role of religious beliefs in influencing hygiene practices (17–19). Similarly, the development of modern infection control has emphasized the scientific foundations of hand hygiene, particularly since the emergence of the germ theory (15, 16). However, these strands of scholarship have largely been examined in separately. Studies from behavioral and public health perspectives have also extensively investigated determinants of handwashing compliance, including knowledge, attitudes, environmental conditions, and intervention strategies (6, 20–22). While these approaches have generated important evidence for improving hand hygiene behaviors, they have generally focused on immediate determinants of individual action rather than the historical processes through which handwashing acquired its contemporary meanings and institutional forms. There remains a lack of integrative frameworks capable of explaining how cultural, religious, and scientific logics co-evolved and how their interactions continue to influence present-day handwashing practices.

Addressing this limitation requires an analytical approach that can account for both the historical development of practices and the social relationships through which these practices are maintained. Cultural-Historical Activity Theory (CHAT) provides such a framework by viewing human activities as historically evolving systems rather than isolated individual behaviors. To address this gap, this study applies CHAT as an analytical lens to conceptualize handwashing as the outcome of interacting activity systems (23). CHAT originates from sociocultural theories of human development and emphasizes that human actions are mediated by cultural tools, social rules, and collective forms of activity (24–26). Within this framework, an activity system refers to a structured network of interconnected elements, including subjects (individuals or groups engaged in an activity), objects (the goals or purposes that motivate the activity), mediating tools (physical or symbolic resources used to achieve the object), rules (social norms and regulations), community (social groups involved in the activity), and division of labor (the distribution of roles and responsibilities among participants). These elements interact dynamically and may generate tensions or contradictions that drive changes in the activity system over time. CHAT provides a means to examine how practices are shaped by the dynamic relationships between actors, tools, rules, and social contexts, while also highlighting the tensions and contradictions within and between systems. In this study, we identified three historically constituted activity systems (cultural, spiritual-religious, and medical-scientific) that have jointly contributed to the development of handwashing practices. By conceptualizing these domains as interconnected activity systems, CHAT enables an examination of how different forms of knowledge and social expectations have interacted, competed, and transformed throughout history (27, 28). This perspective allows handwashing to be interpreted not merely as a behavioral outcome requiring individual modification, but as a historically situated practice embedded within broader social and cultural structures.

By tracing the evolution of these systems from ancient civilizations to the present, this study aims to demonstrate that contemporary challenges in handwashing—such as inconsistent adherence and persistent behavioral barriers—can be better understood as outcomes of misalignments between historically embedded systems rather than as simple deficits in knowledge or resources. This perspective shifts the focus from individual behavior change to a more comprehensive understanding of the social and historical dynamics that shape hygiene practices.

Ultimately, this study contributes to the literature by offering a theoretically grounded, integrative account of handwashing as a socially co-produced practice and highlighting the implications of this perspective for designing more context-sensitive and effective public health interventions.

## 2 Methods

This study employed a theoretical review based on historical evidence. Rather than conducting a systematic review or empirical investigation, it synthesized historical sources through the analytical lens of CHAT. The objective of this study was to examine the historical transformation of handwashing practices by analyzing the interactions among three historically constituted activity systems: (1) the cultural-scientific belief system, (2) the spiritual–psychological wellbeing system, and (3) the medical–scientific system. Specifically, this study aimed to explore how these systems developed, interacted, and transformed over time, and how their contradictions contributed to the emergence of contemporary handwashing practices.

The Behavior Centered Design (BCD) approach to handwashing (29) was considered as a complementary conceptual perspective rather than as the primary methodological frameworks. BCD emphasizes that hygiene behaviors are influenced not only by individual knowledge and motivation but also by external contextual factors, including social, environmental, and institutional conditions. In this study, BCD was used to contextualize the behavioral dimensions of handwashing, whereas CHAT served as the primary analytical framework for examining the historical and sociocultural development of these behaviors. Thus, BCD helped identify relevant behavioral determinants, while CHAT enabled analysis of how such determinants became embedded within historically evolving activity systems.

Specifically, historical and cultural dimensions, such as handwashing-related policies, and local and national leadership on hygiene practices, are considered critical external determinants. These factors were examined as components of broader activity systems rather than as isolated determinants of individual behavior. They provide a structured lens through which the evolution of such behaviors can be systematically conceptualized (30).

**Figure 1:**
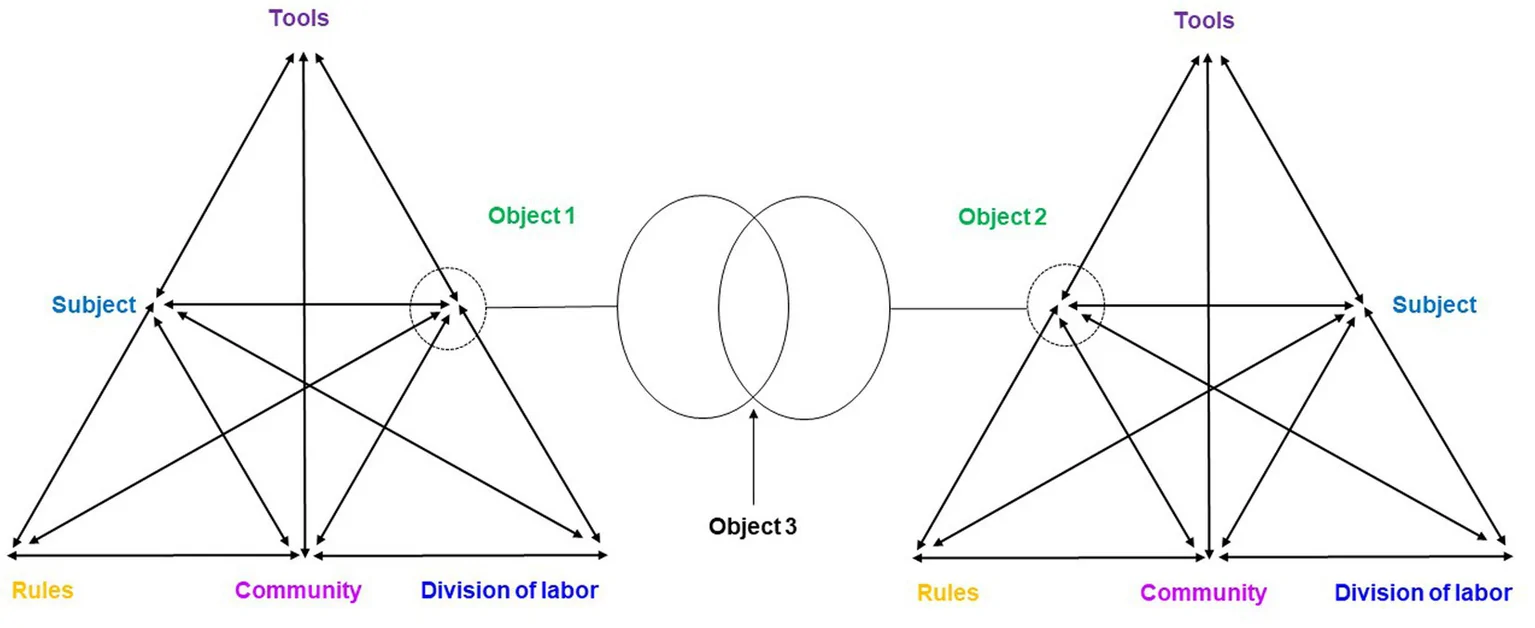
Third generation of CHAT adapted by Engeström, (23).

To capture these dynamics, this study employed a historical research design framed by CHAT. This approach is particularly appropriate given that the history of handwashing cannot be examined in isolation from other factors. Rather, it is deeply intertwined with broader sanitation infrastructure and related practices, including bathing, soap use, and towel use, all of which reflect diverse socio-cultural patterns and material conditions. Accordingly, the cultural and historical contexts are modeled as activity systems. As illustrated in Figure 1, this study utilizes the third-generation CHAT framework developed by Engeström (23).

The analytical process was organized according to three historically interacting activity systems identified through the CHAT framework. First, the cultural-scientific belief system was examined to explore how cleanliness, social norms, environmental adaptation, and cultural knowledge shaped early handwashing practices from ancient civilizations to the Enlightenment. Second, the spiritual–psychological wellbeing system was analyzed to examine how religious traditions incorporated handwashing into moral purification, ritual practice, and collective behavioral regulation. Third, the medical–scientific system was examined to trace the transformation of handwashing into an evidence-based practice through the development of medical knowledge, germ theory, and modern infection control. The findings of the theoretical review are presented thematically in the following sections according to the three historically interacting activity systems identified through the analysis.

## 3 Results

The following sections present the historical evolution of handwashing within each of the three activity systems identified through the CHAT analysis. Although these systems are discussed separately to facilitate analysis, they should be understood as historically interconnected rather than independent. Their interactions, complementarities, and contradictions are synthesized in the final section to explain the evolution of contemporary handwashing practices. Representative historical examples are presented to illustrate the evolution of each activity system rather than to provide an exhaustive historical account.

### 3.1 Cultural-scientific belief system for handwashing: from ancient civilizations to the enlightenment

Health-related behaviors are shaped by multilevel determinants operating at the individual, interpersonal, institutional, community, and policy levels (31). Culturally embedded factors have repeatedly demonstrated their influence on health outcomes across different settings, with successful applications of culturally informed health strategies documented in multiple contexts (32, 33). Culture-focused health interventions are therefore central not only to disease prevention but also to broader health system transformations (17). However, such efforts often face structural constraints, including fragmented political institutions, resistance from vested interests, and fiscal limitations (34), highlighting the necessity of culturally sensitive and context-specific approaches (35).

From a research perspective, culture should not be treated merely as an intervention tool but as a fundamental determinant of health behavior (18). Accordingly, examining the interaction between culture and health requires a deeper historical and systemic analysis of how cultural meanings, material conditions, and institutional arrangements shape behavioral practices. Given that culture is a key social determinant of health (36, 37), understanding its historical evolution is essential for explaining contemporary hygiene behaviors, including handwashing.

Within this framework, handwashing practices are conceptualized as historically evolving components of sociocultural activity systems rather than isolated individual behaviors. This aligns with the theoretical orientation of this study, which integrates BCD and CHAT, emphasizing the role of the external context in shaping behavioral determinants and transformations.

#### 3.1.1 Ancient foundations of cultural-scientific belief systems

Early civilizations developed distinct cultural and scientific belief systems that linked health, cleanliness, and cosmological or spiritual understandings of the body. Civilizations such as Mesopotamia and Ancient Egypt, located along major river systems (38, 39), benefited from abundant water sources that supported both survival and early washing and cleansing practices. However, the emergence of hygiene-related behaviors was not solely driven by material conditions but also by culturally constructed beliefs about health, illness, purity, and spirituality (40). These belief systems differ significantly from modern biomedical paradigms and are shaped by culturally relative understandings of health (41).

In Mesopotamian societies, hygiene practices were closely associated with religious taboos and ritual purity. Priests were required to perform cleansing rituals, and washing practices were embedded in their ceremonial obligations (42). The Code of Hammurabi includes references to purification during epidemics, indicating the early institutionalization of hygiene norms (43). Similarly, Babylonian knowledge of soap-like substances and cleansing mixtures reflects early technological engagement with hygiene (44), while ritual washing practices during festivals further demonstrate the integration of hygiene into cultural and religious systems (45). The ancient Egyptian civilization incorporated regular bathing and hand cleansing into both daily life and medical practice, reinforcing cleanliness as a part of health maintenance (46–48).

Other ancient civilizations further advanced these developments. Minoan and Greek societies, further advanced water infrastructure and public bathing systems (49, 50). While these developments improved sanitation, they also reflected emerging conceptual linkages between environment, health, and bodily cleanliness, as articulated by early thinkers such as Hippocrates (51). The Indus Valley Civilization demonstrated sophisticated urban sanitation systems and ritual bathing practices (52, 53), which later influenced Ayurvedic traditions that emphasized holistic health principles (54). In ancient China, health was conceptualized through cosmological frameworks, such as yin–yang and the five elements (55). Hygiene practices, including frequent handwashing, have been integrated into daily routines and supported by the development of sanitation technologies and cleansing materials (56, 57). These practices also contributed to the linguistic and cultural codification of hygiene-related behaviors (58).

#### 3.1.2 Continuity and transformation in medieval and early modern systems

During the medieval period in Europe, hygiene practices were shaped by structural constraints, such as overcrowding, limited sanitation infrastructure, and widespread disease exposure (59). Religious interpretations of cleanliness also shifted, with public bathing gradually being reframed as a moral or charitable practice rather than a social necessity (60). Nevertheless, handwashing practices persisted in both domestic and elite contexts, often reinforced through texts, visual culture, and emerging social norms (61, 62).

Simultaneously, knowledge about soap production and hygiene continued to evolve, although access remained limited and socially stratified (63, 64). Medical writings and early public health observations, such as those by Gilbertus Anglicus, reflected increasing awareness of personal hygiene, although systemic improvements remained limited (65, 66).

The Renaissance period marked a conceptual shift in hygiene understanding, where bodily cleanliness became increasingly associated with physiological processes and health maintenance rather than purely ritual or moral dimensions (67, 68). This period also saw broader cultural and intellectual transformations that influenced hygiene practices across Europe and beyond (69).

#### 3.1.3 Enlightenment and the emergence of proto-scientific hygiene rationality

During the Enlightenment, improvements in sanitation practices emerged alongside a limited understanding of the microbial causation of disease (70). Although hygiene behaviors were not yet grounded in germ theory, cleanliness became increasingly associated with virtue, discipline, and rationality, particularly among certain religious and social groups (71). By the late Enlightenment period, early empirical observations and medical practices began to recognize the health implications of cleanliness, thus laying the groundwork for modern hygiene science. Nevertheless, handwashing remains unevenly practiced across populations due to limited institutional enforcement and scientific understanding.

#### 3.1.4 Cross-cultural integration and systemic evolution

Across Asia and Eurasia, medical traditions such as naturopathy, Unani medicine, and Siddha systems have emphasized hygiene as part of holistic health approaches (72–74). These traditions influenced regional practices, including in Mongolia, where Buddhist and indigenous medical knowledge integrated hygiene-related practices such as the use of natural cleansing agents and herbal substances (75, 76).

Mongolian nomadic practices also demonstrate adaptive hygiene strategies shaped by environmental constraints, including the use of snow for washing during winter and reliance on water-efficient cleansing methods (77). These practices illustrate how environmental, cultural, and technological factors co-evolve in activity systems.

#### 3.1.5 Handwashing as an evolving activity system

Taken together, the historical trajectory of civilizations demonstrates that handwashing practices have continuously evolved through the interaction of cultural beliefs, environmental constraints, technological development, and institutional structures. Rather than a linear progression, this evolution reflects multiple overlapping activity systems shaped by context-specific meanings and material conditions of the time.

**Figure 2:**
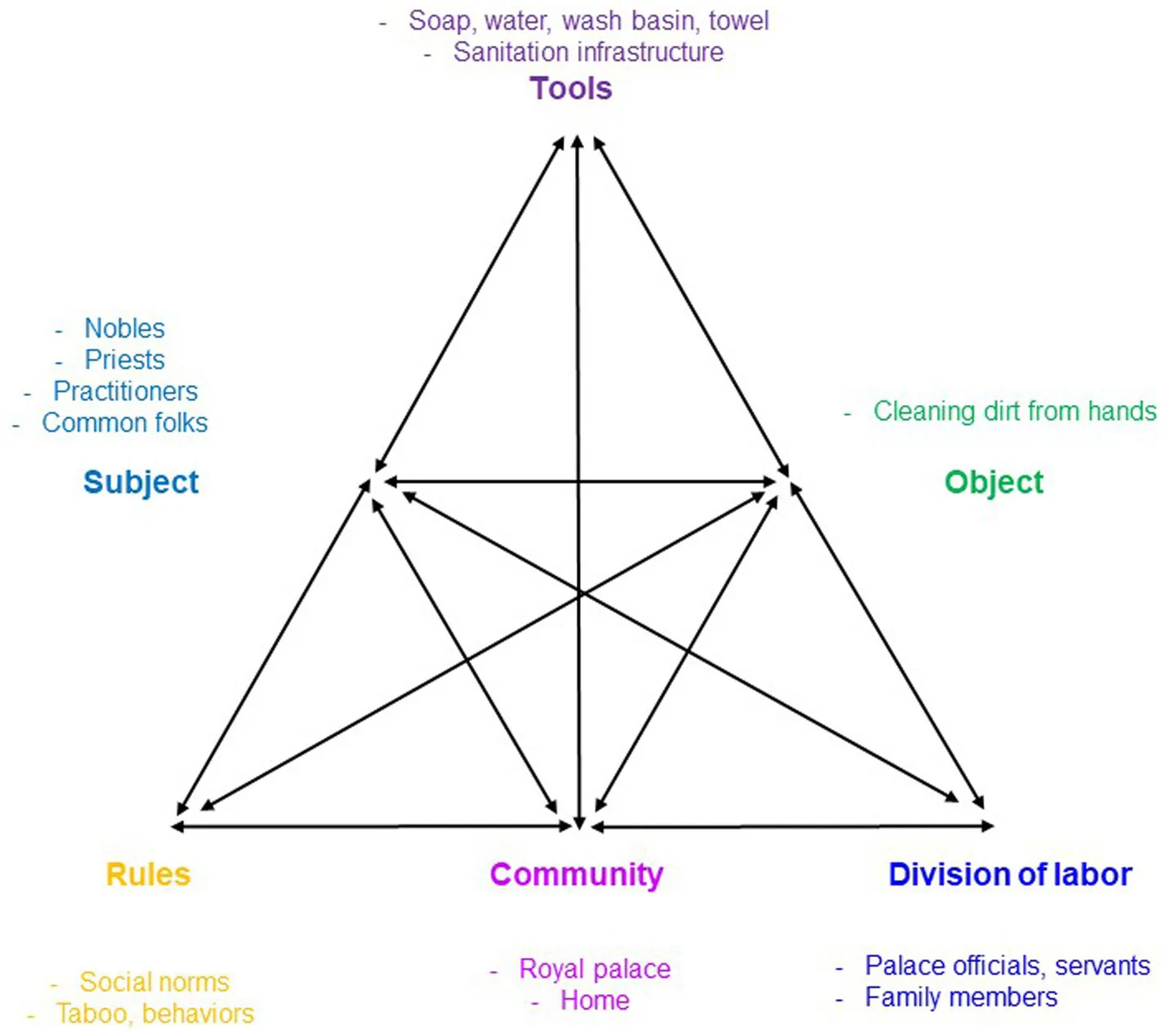
CHAT analysis of cultural-scientific belief system for handwashing. This model enables the analysis of interactions between multiple activity systems, there by offering a more comprehensive understanding of how hand washing practices have evolved in complex sociocultural environments.

Accordingly, this study conceptualizes handwashing as a dynamic cultural-historical activity system, as illustrated in Figure 2, providing a structured framework for analyzing how cultural and scientific belief systems have shaped hygiene practices across time and space.

Figure 2 illustrates handwashing as a historically evolving, object-oriented activity system rather than an individual-level behavior alone. In line with the third-generation CHAT model (23), the system is conceptualized as a dynamic interaction between subjects (individuals and communities), objects (health, cleanliness, and purification), mediating artifacts (water, soap, rituals, and sanitation infrastructure), rules (religious norms and cultural taboos), community (households, religious institutions, and states), and the division of labor (priests, healers, households, and public authorities). Across civilizations, the evolution of this system has been shaped by systemic contradictions, including tensions ritual purity and empirical hygiene practices, between limited water/sanitation infrastructure and normative expectations of cleanliness, and religious authority and emerging scientific explanations of disease. These contradictions have acted as drivers of transformation, periodically reshaping both hygiene practices and the institutional arrangements that sustain them, thereby producing nonlinear pathways in the historical development of handwashing behavior.

### 3.2 Spiritual–psychological wellbeing system for handwashing: analysis of evidence in religious traditions

Handwashing is not merely a modern, science-based construct; historical evidence from both Eastern and Western civilizations demonstrates that it has long been embedded within religious traditions and ritual practices. Across centuries, religious systems have institutionalized handwashing within structured sequences of behavior, assigning it symbolic, moral, and practical significance (19).

From a public health perspective, examining the religious traditions of handwashing is not only historically informative but also practically relevant. Religious beliefs can influence both community-level hygiene promotion and healthcare workers’ compliance with hand hygiene practices (19). More broadly, religion shapes individual understandings of health, illness, and the body (78), and thus represents an important determinant that can be leveraged in hand hygiene promotion strategies (79) and in shaping attitudes toward healthcare and disease prevention (80). Consequently, incorporating belief systems into health policy and program design enhances their contextual relevance and effectiveness (81).

At the conceptual level, religion can be understood as a source of moral meaning in experiences of illness, suffering, and healing (82, 83). This expands the analytical scope of handwashing beyond hygiene to the domain of moral practice and wellbeing. In this regard, handwashing practices intersect with psychological wellbeing (84, 85), particularly given the established association between religiosity and psychological wellbeing (86). Evidence from multiple religious traditions further shows that handwashing serves both symbolic/ritual and hygienic/cleansing purposes (12), as shown in Table 1.

**Table 1:** Specific indications for hand hygiene according to the most widely represented religions worldwide (According to Allegranzi (8)).

Religion	Specific indications for hand hygiene	Reason/purpose
Buddhism	After each meal	Hygienic/cleansing
	To wash the hands of the deceased	Symbolic
	At the new year, young person pour water over elders’ hands	Symbolic
Christianity	Before the consecration of bread and wine	Ritual
	After handling holy oil (Catholics)	Hygienic/cleansing; ritual
Hinduism	During worship (puja) (water)	Ritual
	End of prayer (water)	Ritual
	After any unclean act (toilet)	Hygienic/cleansing
Islam	Repeating ablutions at least 3 times with running water before prayers (5 times/day)	Ritual
	Before and after any meal	Hygienic/cleansing
	After going to the toilet	Hygienic/cleansing
	After touching a dog, shoes, or a cadaver	Hygienic/cleansing
	After handling anything soiled	Hygienic/cleansing
Judaism	Immediately after awakening in the morning	Hygienic/cleansing
	Before and after each meal	Hygienic/cleansing
	Before praying	Ritual
	Before the beginning of Shabbat	Ritual
	After going to the toilet	Hygienic/cleansing
Orthodox	After putting on liturgical vestments before the ceremony	Ritual
Christian	Before the consecration of bread and wine	Ritual
Sikhism	Early in the morning	Hygienic/cleansing
	Before every religious activity	Ritual
	Before cooking and entering the community food hall	Hygienic/cleansing
	After each meal	Hygienic/cleansing
	After taking off or putting on shoes	Hygienic/cleansing

#### 3.2.1 Emergence of religious handwashing practices

Historical evidence further demonstrates that handwashing practices associated with religious traditions were documented across numerous historical societies, although their forms and meanings varied across religious, cultural, and historical contexts. Representative examples from Ancient Egypt, Greece, and Rome, illustrate that ritual handwashing was closely associated with religious observance and ceremonial practice (10, 64, 87, 88).

Religious texts provide more an explicit codification of these practices. In several Hindu traditions, daily purification practices are including bathing and hand cleansing, are described as components of the five Nitya karmas (daily duties), although specific interpretations and practices vary among texts, regions, and schools of thought. Some Dharmaśāstra texts describe omission of certain daily observances as carrying religious consequences (64, 89). Similarly, narratives from Greek mythology, have been interpreted as illustrating how handwashing was functioned within broader systems of ritual purity and moral symbolism (9, 90).

The Hebrew Bible contains passages interpreted as prescribing ritual washing before approaching sacred spaces, with ritual consequences described for noncompliance within those textual contexts. Initially, these practices emphasized washing in situations considered “unclean.” However, over time, the distinction between “unclean” and “impure” became blurred, leading to a generalized rule that all hands must be washed before engaging with sacred objects (91, 92). This gradual shift reflects the transformation from situational to generalized behavioral norms.

Jewish traditions further developed highly structured handwashing practices that were maintained over centuries and, in some cases, contrasted with the surrounding cultural practices (93, 94). During the 14th-century plague, some historical accounts have suggested that certain Jewish communities experienced comparatively lower mortality, although the reasons remain debated and cannot be attributed solelys to their hygiene practices (95, 96). However, this relative resilience also generated suspicion and persecution, illustrating how effective hygiene behaviors can paradoxically produce negative social consequences (97, 98).

In Islamic traditions, ablution (wudu) includes repeated washing of the hands as one component of ritual purification performed before the prescribed prayers. The frequency and manner of practice may vary according to jurisprudential schools and local customs. The Qur’an emphasizes ritual purity and cleanliness, which Islamic scholars have interpreted as providing the foundation for ablution practices (99–103). In Buddhism, monastic codes contain guidance regarding regular washing, cleaning, and bodily maintenance as part of monastic discipline, although practices differ among Buddhist traditions (104). Similarly, Buddhist texts describe the practices related to washing and cleanliness before and after meals and rituals, although their interpretation and implementation vary across historical traditions (105–108).

#### 3.2.2 Contradictions within the spiritual–psychological system

Analyzing these developments through the lens of CHAT reveals several key systemic contradictions that have shaped the evolution of handwashing practices:

1. Ritual vs. Hygienic Function.

Handwashing in religious contexts serves both symbolic (purification of sin, moral cleansing) and practical (removal of dirt or contaminants) purposes. However, these functions are not always aligned with each other. In many cases, ritual compliance does not guarantee hygienic effectiveness, highlighting the tension between meaning and function. For example, the findings showed that ritual handwashing in Jewish and Islamic traditions was primarily intended to achieve ritual purity and fulfill religious obligations. In contrast, the medical scientific system reconceptualized handwashing as a practice aimed at preventing disease transmission. Although the physical act of washing the hands remained similar, the object and purpose of the activity changed across historical contexts. This finding illustrates how the same practice can acquire different meanings and functions within different activity systems.

1. Belief-Based Practice vs. Scientific Knowledge.

Religious motivations for handwashing often emerge independently of scientific understanding of disease transmission. For instance, practices driven by fear of spiritual impurity or divine punishment (e.g., “Macbeth effect”) may have incidentally reduced infection risk, despite lacking biomedical rationale. This creates a contradiction between epistemological systems: belief versus science.

1. Normative Enforcement vs. Social Consequences.

Highly structured hygiene practices within certain religious groups (e.g., communities during medieval plague outbreaks) contributed to lower disease transmissions. However, this divergence also triggered social tensions, including discrimination and persecution (97). Thus, effective practices paradoxically produce negative societal outcomes.

1. Resource constraints vs. ritual expectations

Many religious prescriptions assume access to clean water and purification materials. In situations where water is unavailable or its use is restricted, several religious traditions recognize alternative purification practices. For example, Islam permits dry ablution using clean earth under specified conditions, illustrating how religious practice accommodates environmental constraints while maintaining ritual obligations (109, 110). Rather than representing a departure from religious principles, these adaptations demonstrate the flexibility of ritual systems in responding to material and environmental limitations (13, 111).

#### 3.2.3 Integration and transition toward scientific framing

Despite these contradictions, religious traditions played a crucial role in stabilizing and transmitting handwashing behaviors across generations. They transformed hygiene-related actions into socially reinforced, morally grounded routines, thereby increasing their frequency and consistency.

Importantly, these systems laid the behavioral and cultural foundations upon which later scientific understanding of hygiene could be built. Although the underlying motivations differed, the routinization of handwashing in religious contexts created conditions conducive to its eventual reinterpretation within biomedical frameworks.

**Figure 3:**
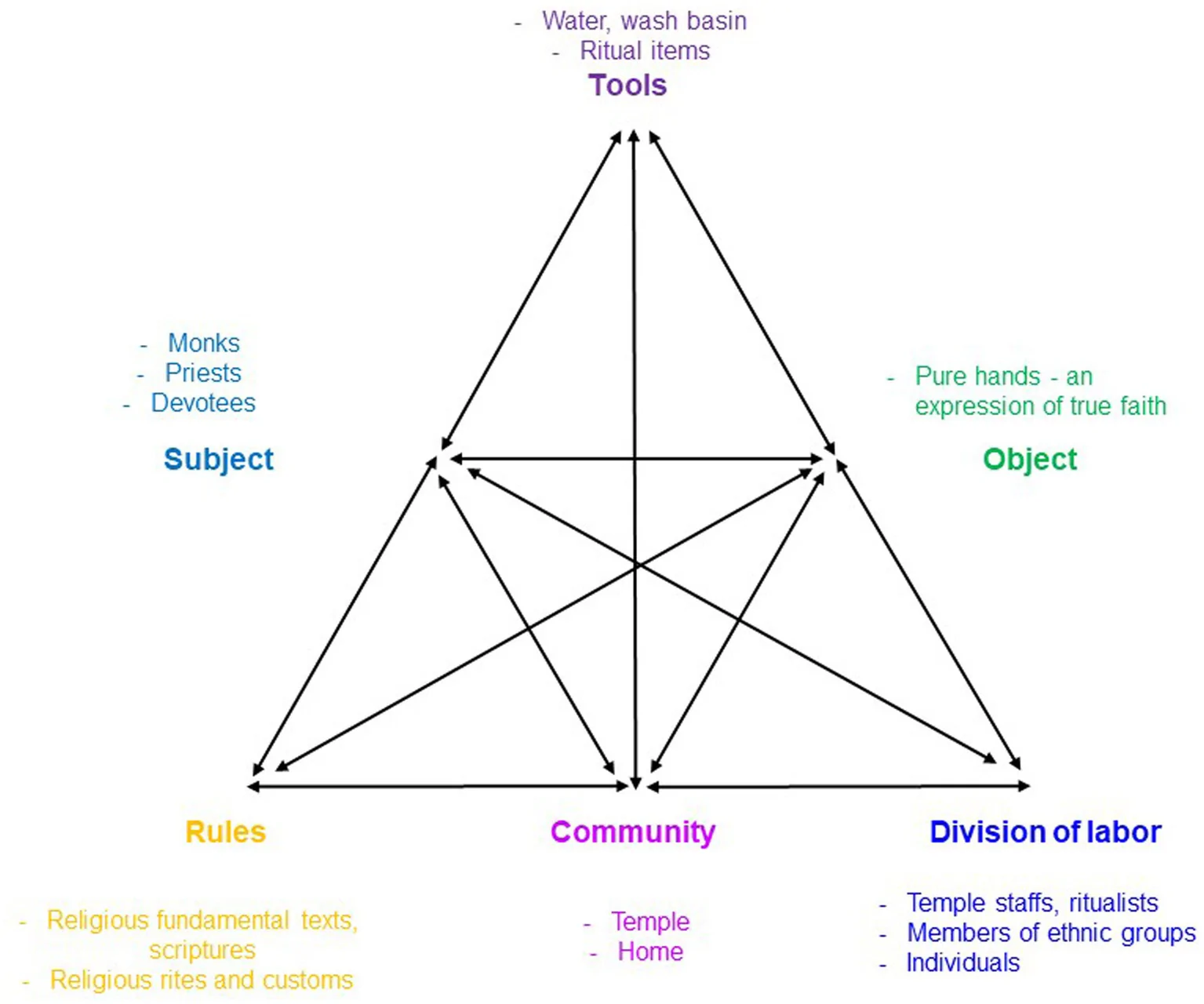
CHAT analysis of spiritual-psychological wellbeing system for handwashing.

From a CHAT perspective, these dynamics can be conceptualized as a spiritual–psychological wellbeing activity system, where handwashing is oriented toward moral purification and wellbeing. As illustrated in Figure 3, the system consists of interacting elements—subjects (believers), objects (purification and wellbeing), tools (water and ritual practices), rules (religious doctrines), community (faith groups), and division of labor (religious authorities and practitioners). The contradictions within and between these elements act as drivers of transformation, enabling the evolution of handwashing practices across historical contexts and facilitating their eventual alignment with broader health and hygiene framework.

### 3.3 Medical–scientific system for handwashing: analysis of the work of famous thinkers and researchers

The medical and scientific understanding of handwashing did not emerge suddenly but evolved gradually through the contributions of philosophers, physicians, and researchers across several centuries. Unlike the cultural and religious systems discussed in the previous sections, this system increasingly sought to explain handwashing through observation, reasoning, and eventually empirical evidence. However, this transition was neither linear nor contested. Instead, it was shaped by the continuous tensions between emerging scientific insights and the dominant paradigms of the time.

#### 3.3.1 Early integration of moral, religious, and medical reasoning: Maimonides

Maimonides (1138–1,204), a prominent medieval philosopher and physician, represents an early attempt to integrate religious doctrine with medical reasoning. His writings include explicit references to handwashing and hand-drying practices (112), positioning him as one of the earliest contributors to handwashing within a medical framework. In Mishneh Torah, handwashing is categorized among the five positive commandments of rabbinic origin (113), reflecting its strong moral and religious grounding. At the same time, Maimonides advised physicians to wash their hands after contact with patients, emphasizing a practical dimension: “Never forget to wash your hands after having touched a sick person” (112). However, this dual positioning also reveals a fundamental contradiction: while handwashing was recognized as important, it remained embedded within religious and even magical interpretations (114), limiting its development as a purely medical practice. Consequently, his ideas were not widely adopted by contemporary medical systems.

#### 3.3.2 Conceptualizing disease transmission: Girolamo Fracastoro

Girolamo Fracastoro (1478–1,553) introduced a major conceptual shift by proposing that diseases are transmitted by invisible “spores” or particles. His 1,546 work represented a significant departure from the dominant miasma theory and laid the foundation for the later germ theory (115). Fracastoro distinguished between direct, indirect, and airborne transmissions (116), providing an early theoretical framework for understanding contagion. Despite its significance, his theory illustrates a contradiction between theoretical innovation and social acceptance of the theory. The concept of invisible particles was difficult for contemporaries to accept (117), and therefore did not translate into widespread behavioral change, including handwashing practices.

#### 3.3.3 Recognizing physicians as vectors: Gordon and pre-Semmelweis thinkers

Alexander Gordon (1752–1799) made the critical observation that physicians themselves could act as vectors of infection, particularly in cases of puerperal fever (118). He explicitly rejected the miasma theory and argued that infection was transmitted through contact, recommending hand and clothing hygiene for healthcare providers (119). Similarly, earlier physicians, such as John Burton, suggested related ideas, although without fully explaining causation (9). Collectively, these contributions represent an important shift toward recognizing human-mediated transmission. However, these insights reveal a contradiction between the emerging empirical observations and the entrenched theoretical frameworks. Despite recognizing the transmission pathways, the absence of a clear biological mechanism limits its acceptance and implementation.

#### 3.3.4 Early practical guidelines without adoption: Zsoldos János

Zsoldos János (1767–1832) provided one of the earliest explicit written guidelines recommending handwashing with soap and water for physicians and midwives (120). His recommendations were based on practical experience, including work in military hospitals during the Napoleonic War (9). Although his work gained recognition, it was not widely adopted at the time. This reflects a contradiction between the available practical solutions and the lack of systemic uptake, a recurring theme in the history of handwashing.

#### 3.3.5 Chemical innovation and antisepsis: Labarraque

Antoine Germain Labarraque (1777–1850) advanced hand hygiene through the introduction of chemical disinfection methods, particularly chlorinated solutions. His work demonstrated that such solutions could reduce odor and potentially prevent infection (121). Labarraque’s contribution marked a transition from simple washing to chemical antisepsis, significantly expanding the “tools” available within the activity system (2, 122–124). However, this stage also reflects a contradiction between technological innovation and conceptual understanding, as antiseptic methods were introduced before the full acceptance of microbial causation.

#### 3.3.6 Advocacy without immediate impact: Oliver Wendell Holmes

In 1843, Oliver Wendell Holmes (1809–1894) argued that healthcare workers could transmit puerperal fever if they failed to wash their hands and clothing (125). He recommended strict preventive measures, including avoiding patient contact after exposure to infected cases (126). Although his work was later recognized, it initially received limited attention because of publication constraints and a lack of institutional support (127). This highlights a contradiction between evidence generation and knowledge dissemination, where valid insights fail to influence practice because of systemic barriers.

#### 3.3.7 Empirical proof and strong resistance: Ignaz Semmelweis

Ignaz Semmelweis (1818–1865) provided the first statistical evidence linking hand contamination to disease transmission (128–130). He demonstrated that mortality rates from puerperal fever were significantly higher in clinics where physicians did not disinfect their hands after autopsies (131, 132). By introducing chlorinated lime hand disinfection, he achieved dramatic reduction in mortality. However, his findings were met with strong resistance, as they directly challenged prevailing medical beliefs. This represents one of the most striking contradictions in the activity system: clear empirical evidence versus professional rejection and effective intervention versus lack of adoption. This tension illustrates how dominant paradigms can override evidence, thereby delaying critical public health advances.

#### 3.3.8 System-level reform: Florence nightingale

Florence Nightingale (1820–1910) shifted the focus from individual behavior to systemic and environmental reform. During the Crimean War, she implemented hygiene measures including handwashing, sanitation infrastructure, and ventilation which significantly improved health outcomes (133, 134). Her Environmental Theory emphasized that a clean environment, including personal hygiene, is essential for recovery (135). Thus, Nightingale contributed to embedding handwashing within institutional and policy-level practices, aligning closely with the external context emphasized in Behavior Centered Design.

#### 3.3.9 Integration with germ theory and surgical practice: Joseph Lister

Joseph Lister (1827–1912) built upon emerging microbiological knowledge to develop antiseptic surgical techniques. By applying disinfectants and promoting hygiene practices, he significantly reduced the incidence of postoperative infections (136, 137). Lister’s work represents a critical stage where scientific theory, practical tools, and institutional adoption began to align, enabling the widespread acceptance of handwashing in medical settings (138, 139).

#### 3.3.10 Contradictions and system transformation

When analyzed through the framework of CHAT, the medical–scientific system of handwashing reveals several key contradictions that drove its evolution

- Theory vs. Practice: Early theoretical insights (Fracastoro) did not translate into behavioral change.
- Evidence vs. Acceptance: Empirical findings (Semmelweis, Holmes) were resisted by dominant medical communities.
- Innovation vs. Adoption: Practical solutions (Zsoldos, Labarraque) existed but were not widely implemented.
- Individual vs. System-Level Change: Early focus on individual behavior evolved into institutional reforms (Nightingale).
- Knowledge vs. Paradigm: Scientific advances conflict with entrenched beliefs (miasma theory), delaying progress.

These contradictions acted as drivers of transformation, gradually reshaping the system from belief-based and fragmented practices into a coherent, evidence-based framework.

**Figure 4:**
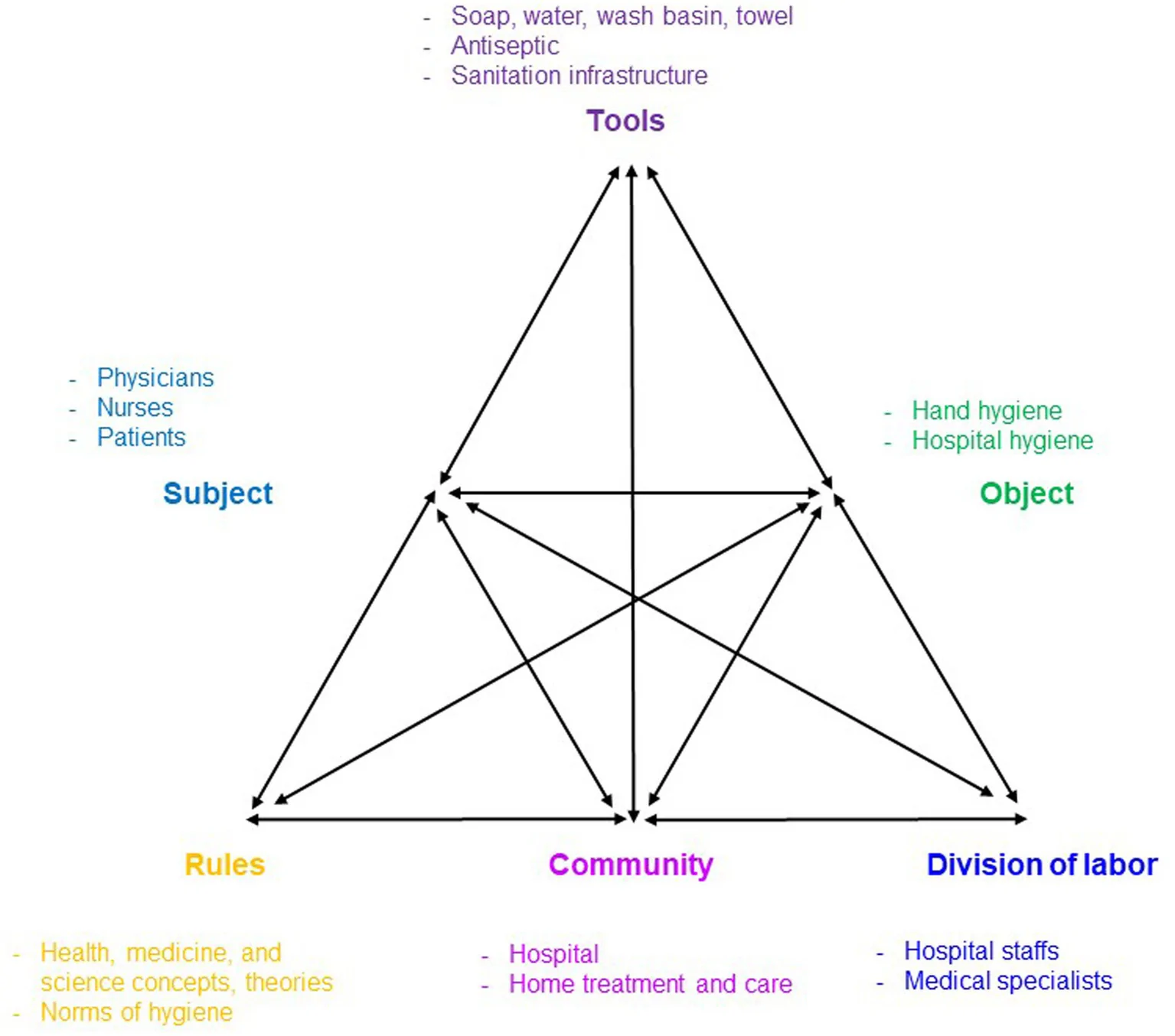
CHAT analysis of medical-scientific system for handwashing.

Thus, the process of understanding, explaining, and promoting handwashing from a medical-scientific perspective has developed over centuries based on the active work of thinkers and researchers. These can be analyzed within an activity system, as illustrated shown in Figure 4.

The medical-scientific system for handwashing can be conceptualized as an activity system in which the subject (physicians, researchers, and healthcare workers); the object is (prevention of disease transmission); the tools (water, soap, antiseptics, and scientific knowledge); the rules (medical guidelines and professional norms); the community (medical institutions and scientific communities); and the division of labor (researchers, clinicians, and policymakers). As illustrated in Figure 4, the evolution of this system was driven by the resolution of internal contradictions, leading to the eventual consolidation of handwashing as a fundamental practice in modern medicine.

### 3.4 The modern scientific–cultural–religious multifaceted system for handwashing: since the twentieth century

The twentieth century marked a decisive turning point in the history of handwashing, as it became widely recognized as a scientifically validated as a fundamental measure for preventing infectious diseases and was increasingly institutionalized across healthcare, food safety, and public health systems (16, 140). However, this transition was nonlinear. Although handwashing had already become part of everyday life, its scientific rationale was not yet fully understood by the general population, reflecting a continuing gap between practice and scientific knowledge (141).

Infections established the scientific foundation for modern infection. Scientific advances progressively narrowed this gap. Research on bacteria, evidence demonstrating the effectiveness of handwashing in reducing pathogen transmission, and studies on healthcare associated infections established the scientific foundation for modern infection prevention throughout the mid twentieth century (142–144).

From the 1970s onward, successive national and international guidelines standardized hand hygiene practices by defining techniques, indications, and infection prevention strategies (15, 145–147). Despite these advances, implementation studies continued to demonstrate a persistent gap between knowledge and practice, particularly in healthcare settings, highlighting the importance of behavioral and sociocultural factors alongside biomedical evidence (148–150).

In the twenty-first century, handwashing has evolved into a multifaceted system, shaped by interactions among scientific evidence, cultural norms, religious values, and global governance structures.

International initiatives, including Global Handwashing Day and responses to the COVID-19 pandemic, reinforced the importance of sustained hand hygiene while also revealing the continuing need to move from reactive responses toward long term preventive strategies (3, 151). CHAT perspective, this evolution reflects the increasing interaction of scientific, cultural, and institutional systems in shaping contemporary handwashing practices.

However, persistent challenges remain. Compliance with hand hygiene guidelines remains suboptimal in both healthcare and educational settings. This has led to the emergence of innovative approaches, including the application of nudging theory and the integration of artificial intelligence to design adaptive and context-sensitive behavioral interventions (152). These developments reflect an ongoing effort to resolve the enduring contradiction between awareness and consistent behavior.

**Figure 5:**
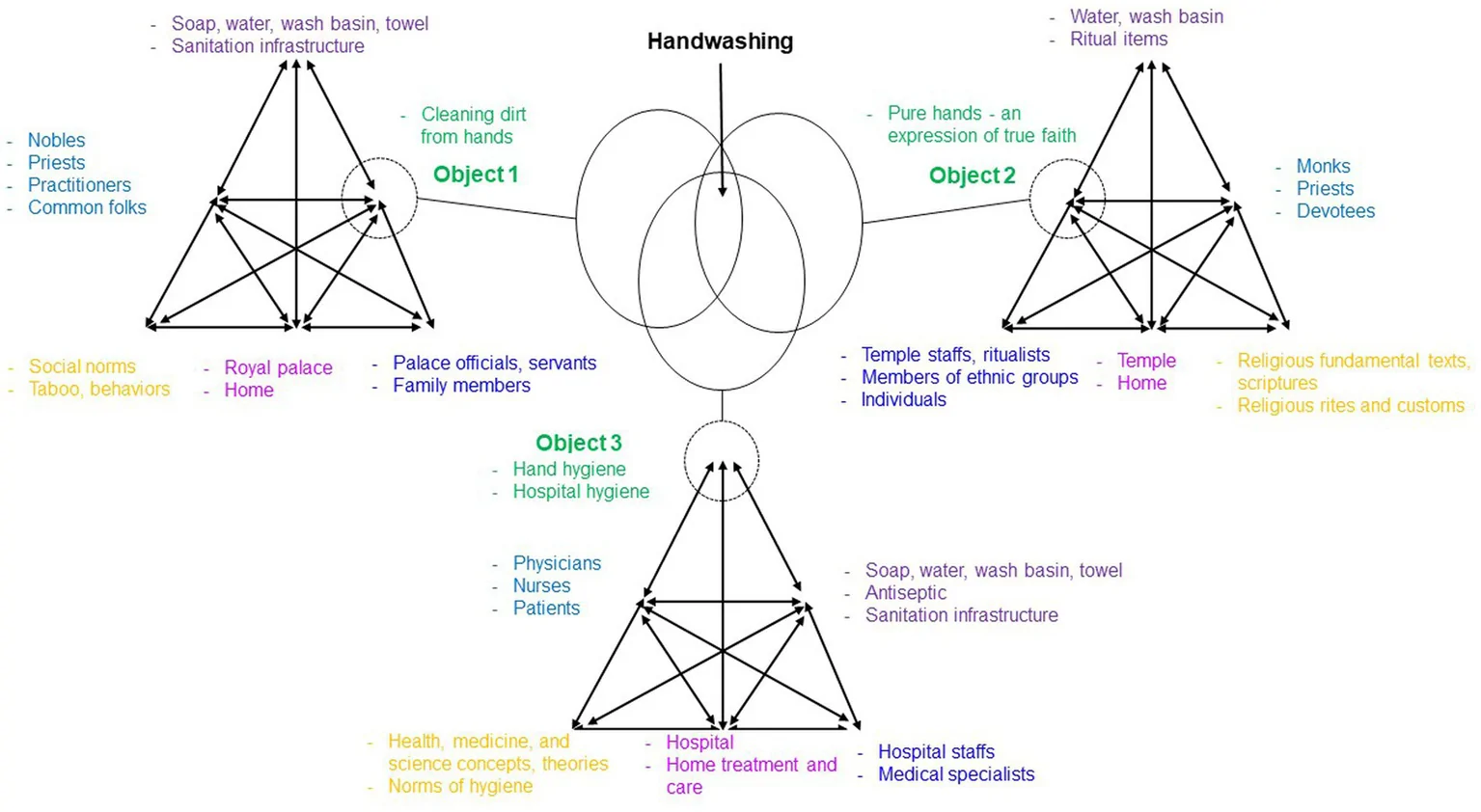
CHAT analysis of scientific-cultural-religious multifaceted system for handwashing.

Figure 5 illustrates the contemporary handwashing system as a dynamic configuration of interacting scientific, cultural, religious, and policy-driven activity systems. Rather than functioning independently, these systems continuously shape and transform each other through multiple layers of contradictions and adaptations.

At the core of this system lies a persistent tension between knowledge and behavior, where scientifically validated practices do not always translate into consistent, everyday actions. This contradiction is further intensified by the coexistence of culturally embedded habits and religiously informed meanings, which may either reinforce or diverge from biomedical recommendations.

From a CHAT perspective, these tensions should not be interpreted as barriers but as drivers of transformation, enabling the evolution of handwashing practices over time. Therefore, the modern system does not replace earlier cultural or religious logics but integrate them into a more complex, multi-layered behavioral structure.

In this sense, handwashing emerges as both a biomedical intervention and a socio-cultural practice, sustained through the interplay of evidence, meaning, norms, and institutional regulations. This integrated system provides a foundation for future innovations, including behaviorally informed interventions and technology-driven solutions, which aim to resolve the enduring gap between awareness and practice.

In conclusion, the evolution of handwashing since the twentieth century demonstrates the emergence of a modern scientific–cultural–religious multifaceted system, in which multiple activity systems intersect and co-evolve. Scientific knowledge provides an evidence base; cultural norms shape everyday practices; religious traditions reinforce moral and symbolic meanings; and policy systems enable large-scale implementation.

Taken together, these historical analyses demonstrate how the interaction among cultural, spiritual–religious, and medical–scientific activity systems has co-produced contemporary handwashing practices. These findings provide the basis for the conclusions presented below.

### 3.5 Implications for policy and practice

The findings of this study suggest that handwashing promotion should extend beyond infrastructure provision and knowledge-based education to incorporate the historical, cultural, and religious contexts that shape hygiene behaviors. Public health interventions may therefore benefit from adopting culturally responsive strategies that acknowledge the values, beliefs, and social norms influencing handwashing practices in different communities.

For ministries of health, these findings highlight the importance of integrating sociocultural assessments into national hand hygiene policies and behavior change programs. Rather than applying standardized messages across all populations, health promotion strategies could be adapted to local cultural and religious contexts while remaining consistent with evidence-based infection prevention principles (153).

Similarly, international organizations and non-governmental organizations involved in water, sanitation, and hygiene (WASH) programs may strengthen intervention effectiveness by collaborating with community leaders, religious organizations, and other local stakeholders. Such partnerships can facilitate the co-design of culturally appropriate handwashing campaigns, improve community acceptance, and support sustainable behavior change (5, 154). The Sustainable Development Goals highlight key accelerators, including governance, financing, capacity development, data systems, and innovation (151), that must operate synergistically rather than in isolation. Within this framework, the integration of cultural and religious systems is not supplementary but essential for achieving sustainable behavioral change.

From a CHAT perspective, these approaches recognize that handwashing behaviors emerge from interactions among multiple activity systems. Consequently, sustainable improvements are more likely to be achieved when interventions address not only individual knowledge and infrastructure but also the broader cultural, institutional, and historical systems within which hygiene practices are embedded.

Looking ahead, advances in hygiene technologies, including sensor-based monitoring systems, improved antimicrobial formulations, and more accessible hygiene solutions, provide new opportunities to strengthen hand hygiene practices. However, the findings of this study suggest that technological innovation alone is unlikely to achieve sustained behavioral change (155). Consistent with the CHAT perspective, effective interventions should combine technological advances with culturally appropriate education, behavioral strategies, and institutional support to promote sustainable hand hygiene practices.

### 3.6 Limitations

Although this study provides a comprehensive historical interpretation of handwashing through the analytical lens of CHAT, several limitations should be acknowledged.

First, this study was based on a theoretical review of historical literature rather than a systematic review or empirical investigation. Consequently, the findings should be interpreted as a conceptual synthesis rather than as a comprehensive historical account of all civilizations or religious traditions.

Second, the historical evidence included in this study was selectively synthesized to illustrate the development and interaction of cultural, spiritual-religious, and medical-scientific activity systems. As a result, certain regional traditions, local practices, and historical variations may not have been fully represented.

Third, interpretations of historical and religious texts inevitably involve contextual and scholarly variation. Although representative primary and secondary sources were used where appropriate, religious practices have evolved across different periods, regions, and denominational traditions. Therefore, the examples presented in this study should not be interpreted as universally representative of all historical or contemporary religious communities.

Finally, CHAT served as the primary analytical framework for interpreting historical developments. While this perspective provides valuable insights into the interactions among activity systems, alternative theoretical approaches may offer complementary interpretations of the historical evolution of handwashing.

Future research could extend this work by conducting systematic historical reviews, comparative cross-cultural analyses, and empirical investigations to examine how historically embedded cultural and religious practices continue to influence contemporary hand hygiene behaviors in different social contexts.

## 4 Conclusion

Handwashing should not be understood solely as a hygienic practice within medical settings. Rather, this study demonstrates that it is a socially and historically co-produced practice shaped through the interaction of cultural, religious, and scientific activity systems.

Using CHAT CHAT as an analytical framework, this study shows that the evolution of handwashing cannot be explained by scientific progress alone. Instead, it reflects the continuing interaction of cultural norms, religious meanings, institutional structures, and scientific knowledge. The analysis further demonstrates that historical contradictions, including those between ritual and hygiene, knowledge and practice, and policy and implementation, have contributed to the ongoing development of contemporary hand hygiene.

Recognizing handwashing as a socially and historically co-produced practice broadens our understanding of its development and provides a stronger theoretical foundation for designing context-sensitive, inclusive, and sustainable public health interventions.
